# Reduction in Liver Cancer Risk by Quercetin via Modulation of Urate Levels: Insights from Drug-Target Mendelian Randomization

**DOI:** 10.3390/genes16040449

**Published:** 2025-04-13

**Authors:** Zhengwen Li, Yue Wang, Kaichuan Yang, Chujie Li, Ming Zhang

**Affiliations:** 1 School of Pharmacy, Chengdu University, 2025 Chengluo Avenue, Chengdu 610106, China; yangkaichuan@cdu.edu.cn; 2 Cell Biology-Inspired Tissue Engineering, Institute for Technology-Inspired, Regenerative Medicine, Maastricht University, 6200 MD Maastricht, The Netherlands; yue.wang@maastrichtuniversity.nl; 3 Precision Medicine, Faculty of Health, Medicine and Life Sciences, Maastricht University, 6200 MD Maastricht, The Netherlands; c.li@maastrichtuniversity.nl; 4 College of Food Science and Engineering, Hainan University, 58 Renmin Road, Haikou 570228, China

**Keywords:** quercetin, urate, liver cancer, drug-target Mendelian randomization

## Abstract

Background: Quercetin, a dietary flavonoid and a widely used supplement, has hepatoprotective properties. Given its urate-lowering effects and epidemiological evidence linking elevated serum urate levels to liver cancer risk, we tested whether quercetin reduces liver cancer risk via modulation of urate levels by bioinformatics methods. Methods: We employed drug-target Mendelian randomization using genome-wide association study summary statistics from public databases (e.g., MRC-IEU) to assess genetic associations, and integrated these findings with GEO datasets (such as GSE138709 and GSE179443) and immune infiltration analyses using tools like xCell, TIMER. Results: Our analyses identified *ABCG2*-mediated urate elevation as a causal risk factor for hepatocellular carcinoma (OR = 1.001, *p* < 0.01), cholangiocarcinoma (OR = 3.424, *p* < 0.01), and liver fibrosis (OR = 2.528, *p* < 0.01). Single-cell transcriptomics revealed elevated *ABCG2* expression in cholangiocarcinoma endothelial cells, while immune infiltration analysis showed significant associations between *ABCG2* expression and both endothelial cell and macrophage infiltration. Survival analysis further indicated that *ABCG2* was not associated with poor prognosis in cholangiocarcinoma or hepatocellular carcinoma. Conclusions: Considering quercetin’s multifaceted interactions with BCRP/*ABCG2*, our findings support its potential use as a preventive dietary supplement for hepatic diseases rather than as an adjunctive therapy for established liver cancer.

## 1. Introduction

Quercetin is ubiquitously distributed across multiple botanical species, including traditional Chinese medicinal herbs such as Ligusticum chuanxiong, Ophiopogon japonicus and Fritillaria cirrhosa [1,2], It is also present in our diet and widely marketed as supplement, has anti-inflammatory, antioxidant, and anticancer properties [3]. However, the rationale for the various health claims of quercetin, the molecular mechanisms underlying its health benefits, remain unclear.

The primary liver cancers are hepatocellular carcinoma (HCC), cholangiocarcinoma (CCA), mixed hepatocellular-cholangiocarcinoma, and pediatric types [4,5]. They are strongly associated with chronic liver diseases. HCC, the most common type of liver cancer (~75–85%), is the third leading cause of cancer-related mortality, primarily due to late-stage diagnosis, limited therapeutic options, and complex pathogenesis [6,7,8]. Investigations by Toru Hisaka et al. [9], have demonstrated that quercetin suppresses HCC, indicating that quercetin might be used for the prevention or treatment of HCC. Additionally, as a therapeutic adjuvant quercetin enhances the efficacy of chemotherapeutic agents such as 5-fluorouracil [10].

Emerging evidence links elevated serum uric acid (SUA) levels to hepatocarcinogenesis [11,12]. Elevated SUA levels may contribute to cancer by inducing oxidative stress, DNA damage, and NLRP3 inflammasome activation, creating a tumor-promoting microenvironment [13,14,15]. Cohort studies further suggest that hyperuricemia is associated with reduced overall survival and increased recurrence in liver cancer patients [16,17]. Quercetin has demonstrated urate-lowering properties via multiple mechanisms. Preclinical models show that it downregulates urate transporter 1 (URAT1) and glucose transporter 9 (GLUT9), leading to reduced SUA levels [18]. Clinical studies also confirm that quercetin supplementation significantly lowers SUA without affecting glycemic control or renal urate excretion [19]. Moreover, epidemiological data from NHANES and FNDDS reveal an inverse correlation between flavonoid intake and both HCC incidence and hepatic fibrosis progression [20].

Given these findings, the hypothesis emerges that quercetin may influence liver cancer progression through urate regulatory mechanisms. To empirically validate this hypothesis, we employed Mendelian randomization (MR) integrated with genomic data on liver diseases from the GEO database.

MR, a genetic epidemiological approach, can be used to establish causal relationships between exposures and disease outcomes using instrumental variables (IVs) derived from single nucleotide polymorphisms (SNPs) [21]. However, conventional MR analyses have largely found no direct causal relationship between SUA levels and cancer, including liver cancer [22]. This may be due to pleiotropy, where SNPs influence multiple traits, confounding causal inference in multi-SNP MR analyses. To address this, we applied drug-target MR, focusing on gene-protein interactions relevant to quercetin’s mechanism of action. Drug-target MR utilizes protein quantitative trait loci (pQTLs) or upstream genetic markers, such as expression quantitative trait loci (eQTLs), to refine causal inference [23]. For instance, studies have successfully used this method to investigate oncogenesis-related drug targets, such as proprotein convertase subtilisin/kexin type 9 (PCSK9) inhibitors and antihypertensive medications [24]. In our study, SUA levels—modulated by quercetin—serve as the exposure phenotype, with cis-acting genetic variants (±100 kb from target loci) selected as IVs, following the target gene identification.

Our study aimed to elucidate the causal role of *ABCG2* genetic variants in modulating urate levels and their impact on liver diseases, including HCC, CCA, and liver fibrosis. We found that these variants mediate elevated SUA levels and significantly increase the risk of these conditions.

## 2. Materials and Methods

### 2.1. Data Source and Preprocessing

The protein targets of quercetin were obtained from the report by Zu et al. [25]. These targets were manually collected or derived from Swiss Target Prediction [26]. The transcriptomic data for liver cancer were downloaded from the HCCDB (http://lifeome.net/database/hccdb2, accessed on 1 January 2025, HCCDB25, HCCDB30) and subjected to a differential expression analysis. By computationally overlaying differentially expressed genes (DEGs) with quercetin-target encoding genes, we identified 11 upregulated and 10 downregulated consensus genes (Supplementary Table S1), including two urate-associated downregulated regulators, ATP-binding cassette subfamily G member 2 (*ABCG2*) and *XDH*. For MR analyses, genome-wide association study (GWAS) summary statistics for hepatic pathologies were retrieved from the MRC-IEU consortium portal (https://gwas.mrcieu.ac.uk/; accessed 1 January 2025), with detailed dataset specifications provided in Table 1.

### 2.2. Drug-Target Mendelian Randomization Analysis

We utilized SNPs located within 100 kilobases of *ABCG2* that are associated with urate, sourced from the GWAS datasets. We selected SNPs with genome-wide significances (*p* < 5 × 10^−8^) and clumped them at linkage disequilibrium (LD) R^2^ < 0.3. This process identified multiple genome-wide significantly associated SNPs based on different results. The selective SNPs can be found in Tables S2–S6. IVW (Inverse Variance Weighted) method [27] was used as the primary analytical method to determine causality. In the MR analysis, we used weighted median (WM) [28] and MR-PRESSO [29] methods to confirm the robustness and reliability of IVW MR estimates. Sensitivity analyses, including a heterogeneity analysis and pleiotropy testing, were conducted using Cochran’s Q test and the MR-Egger method [30], where a *p*-value > 0.05 indicated no heterogeneity. In cases where heterogeneity was detected, a random-effect model was used to replace the IVW results. A *p*-value exceeding the adjusted threshold but remaining below 0.05 was considered to indicate a suggestive association. The MR analysis and sensitivity analysis were performed using the “TwoSampleMR” and “MRPRESSO” packages in R software (version 4.1.1).

### 2.3. Bioinformatics Analysis

We also performed a bioinformatics analysis on the screened upregulated and downregulated genes. Briefly, the identified genes were subjected to Gene Ontology (GO) functional enrichment analysis [31] using the “clusterProfiler” and “enrichplot” packages. The results were ranked based on enrichment thresholds, such as *p*-values. Gene Set Enrichment Analysis (GSEA) [32] was performed to confirm pathway alignment with the GO results. The Xcell [33] and TIMER methods [34] were used to assess the relationship between the expression of the screened genes and immune cell infiltration. The results were visualized using “ggplot2” [35].

## 3. Results

### 3.1. MR Analysis Results Show That ABCG2 Has a Causal Relationship with Urate

**Figure 1:**
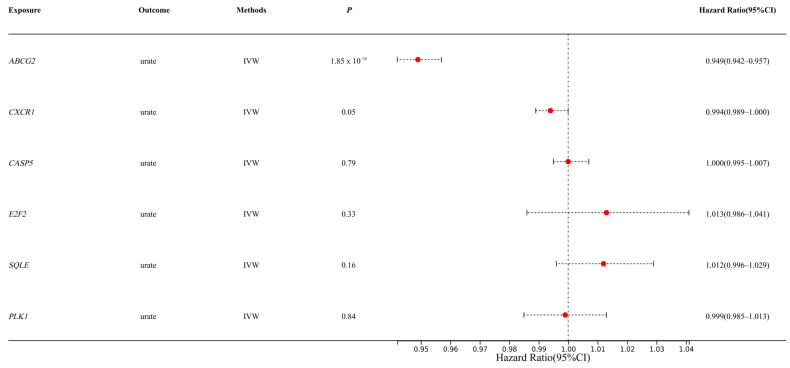
Estimation of causal effects of selected genes on urate levels (IVW: Inverse Variance Weighted method; Hazard Ratio (95% CI) = OR; The Red spots represent the OR values).

We first extracted SNPs from eQTL databases (https://www.eqtlgen.org/cis-eqtls.html, accessed on 1 January 2025) as instrumental variables to evaluate the causal relationship between the selected upregulated/downregulated genes and urate levels. Among the 21 candidate genes, we identified 6 genes with available genetic instruments. The IVW method revealed that only *ABCG2* demonstrated a significant causal association with urate levels (*p* < 0.01) (Figure 1), consistent with the previous reports linking *ABCG2* genetic variants to urate metabolism. The specific SNPs used for each gene are shown in Table S7. Subsequently, we employed a drug-target Mendelian randomization analysis to estimate the causal relationship between *ABCG2* variant-mediated uric acid levels and liver diseases.

### 3.2. MR Analysis Results Show That ABCG2 (Urate Related) Has a Causal Relationship with Liver Cancer

**Figure 2:**
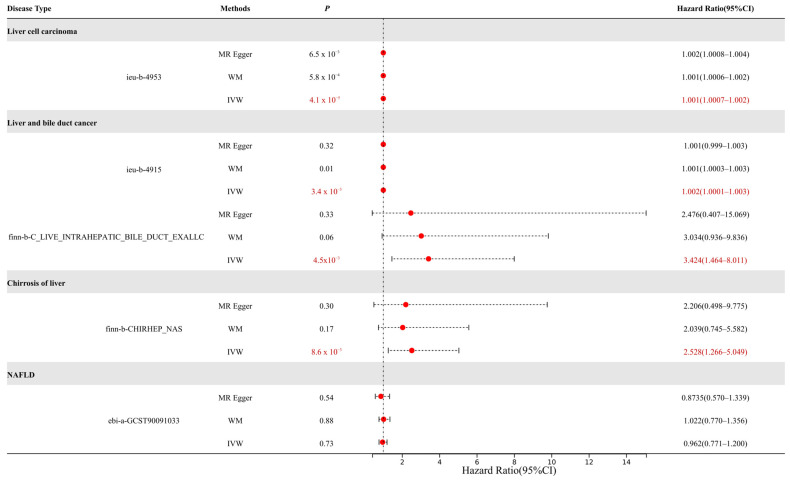
MR results for *ABCG2* expression in liver diseases. The causal relationships that have a *p*-value < 0.05 are marked in red (MR-Egger: Mendelian randomization-Egger; WM: weighted median).

We conducted an MR analysis using several types of liver diseases from the GWAS databases as outcomes, including HCC, CCA, liver fibrosis, and non-alcoholic fatty liver disease (NAFLD) (Figure 2).

The results demonstrated that urate levels mediated by *ABCG2* genetic variants exhibited causal effects on multiple liver diseases. For HCC, the causal effect reached statistical significance (*p* < 0.01) with an odds ratio (OR) of 1.001. For cholangiocarcinoma, both datasets showed significant associations (*p* < 0.01) with OR values of 1.002 and 3.424, respectively. Notably, considering that the dataset (ieu-b-4915) comprised mixed samples of HCC and CCA, we propose that elevated urate levels may play a more critical role in CCA progression compared to HCC. In liver fibrosis, a significant causal relationship was observed (*p* < 0.01, OR = 2.528), indicating hyperuricemia as a risk factor for fibrotic progression. However, no causal association was identified between urate levels and NAFLD.

**Figure 3:**
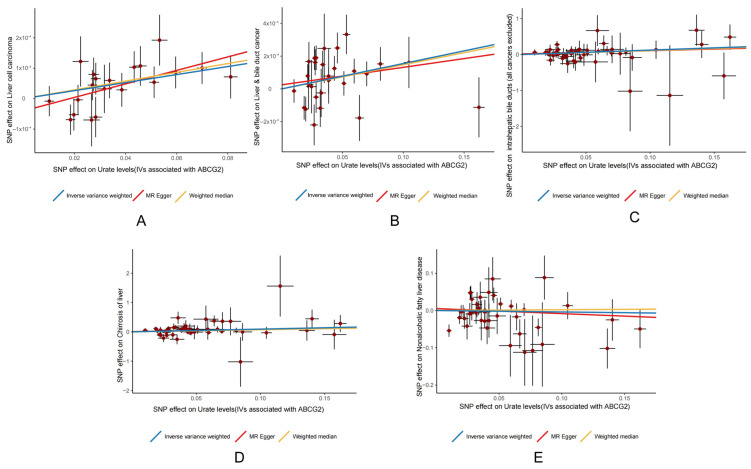
Scatter plots illustrating the relationship between *ABCG2* expression and (**A**) HCC; (**B**) liver and bile duct cancer; (**C**) intrahepatic bile duct cancer (all cancers excluded); (**D**) cirrhosis of the liver; and (**E**) NAFLD. Red spot represent SNPs.

The MR scatterplots depicted the relationships between genetic variants (exposure) and outcomes (Figure 3).

All SNPs closely aligned along the IVW regression line with significant slopes, except for liver and bile duct cancer (Figure 3B) and NAFLD (Figure 3E), indicating that causal estimates were robust for most outcomes.

### 3.3. Heterogeneity and Pleiotropy Analysis

To assess whether the causal effect estimates from the SNPs we used were consistent and to verify whether these SNPs affected the outcomes solely through the exposure factor, we performed heterogeneity tests and horizontal pleiotropy tests (Table 2).

Heterogeneity analysis identified significant heterogeneity in the liver and bile duct cancer dataset (ieu-b-4915) and NAFLD. As a result, random-effect models were applied to these outcomes, confirming a causal relationship between *ABCG2*-mediated hyperuricemia and liver/bile duct cancer (ieu-b-4915), while excluding any such relationship with NAFLD.

Horizontal pleiotropy refers to a gene variant that is associated with more than one phenotype across different biological pathways. To assess this, we used the MR-Egger method to test for horizontal pleiotropy. The *p*-values for liver cell carcinoma, liver and bile duct cancer, cirrhosis of the liver, and NAFLD were 0.181, 0.600 (ieu-b-4915), 0.692 (finn-b-C3_LIVER_INTRAHEPATIC_BILE_DUCTS_EXALLC), 0.840, and 0.607, respectively. As all values were greater than 0.05, this indicates no significant horizontal pleiotropy. The graphical results of horizontal pleiotropy tests can be found in Figure S1.

### 3.4. ABCG2 Expression Is Elevated in CCA Endothelial Cells by Single-Cell Analysis

**Figure 4:**
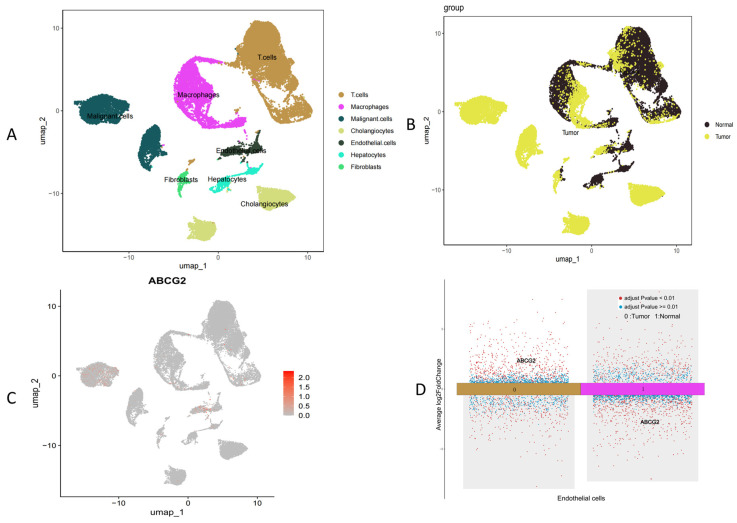
Single-cell RNA sequencing analysis based on GSE138709. (**A**) Clustering of cells by type, (**B**) clustering of cells by normal versus tumor groups, (**C**) *ABCG2* expression across different cell clusters, and (**D**) *ABCG2* expression specifically in endothelial cells.

Given the significant odds ratio (OR) observed between *ABCG2* expression levels and CCA, we examined the differential expression of *ABCG2* in CCA by single-cell RNA sequencing analysis based on GSE138709 (Figure 4).

We found that overall *ABCG2* expression was downregulated in the TCGA (CHOL/CCA) dataset. We found that single-cell analysis (GSE138709) revealed that *ABCG2* expression in specific cells, such as endothelial (Figure 4D) or malignant tumor cells (Figure 4C), was increased compared to the normal group. However, that overall *ABCG2* expression was downregulated in the TCGA (CHOL/CCA) datasets (Figure S2). This discrepancy may be due to different sampling approaches.

### 3.5. ABCG2 Is Associated with Immune Infiltration in CCA

**Figure 5:**
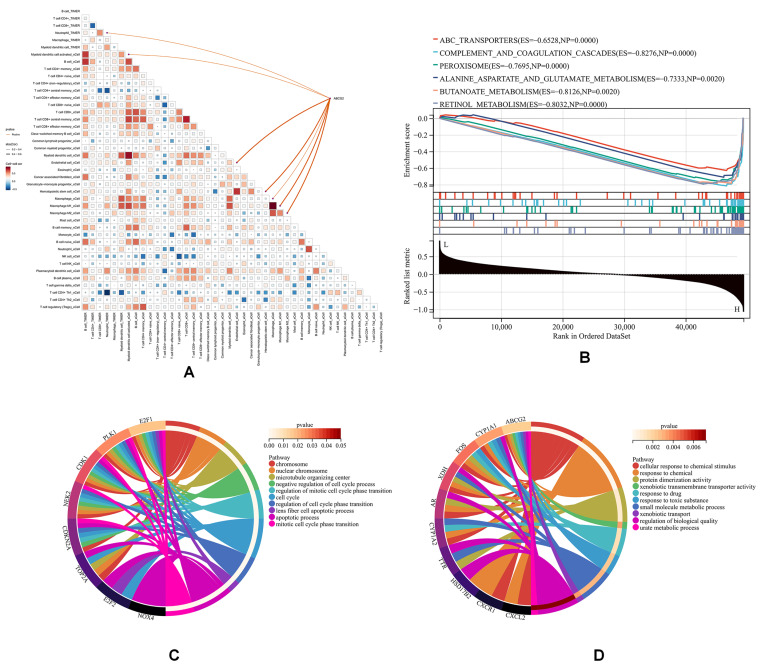
Immune infiltration and GO analysis based on TCGA-CHOL. (**A**) Immune infiltration analysis, (**B**) GSEA using KEGG datasets, (**C**) GO analysis of downregulated genes, and (**D**) GO analysis of upregulated genes.

Given the elevated *ABCG2* expression in endothelial cells of CCA patients, we further investigated its association with immune infiltration (Figure 5).

As shown, *ABCG2* expression exhibited positive correlations with endothelial cell infiltration and macrophage infiltration (*p* < 0.05) (Figure 5A), a trend corroborated by an external cohort (GSE179443, which contained 59 transcriptome profiling cases of iCCA tissue) (Figure S3). GSEA using KEGG datasets revealed that *ABCG2* was particularly involved in drug metabolism and antioxidant pathways (Figure 5B). GO analysis of upregulated (Figure 5C) and downregulated (Figure 5D) genes indicated that downregulated pathways associated with *ABCG2* included “cellular response to chemical stimulus”, “response to toxic substance”, “xenobiotic transmembrane transporter activity”, and “urate metabolic process”. Upregulated genes were mainly enriched in pathways related to “programmed cell death” and “negative regulation of cell cycle processes”, both of which are critically implicated in cancer progression. These findings collectively underscore the multifaceted role of *ABCG2* in modulating both metabolic and immune pathways during hepatobiliary carcinogenesis.

### 3.6. ABCG2 Is Not Associated with Poor Prognosis in CCA

**Figure 6:**
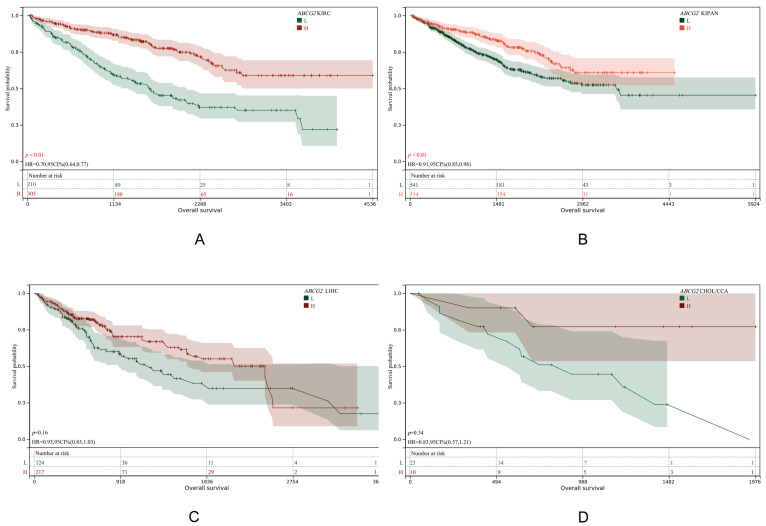
KM plot of *ABCG2* expression in KIRC (**A**), KIPAN (**B**), LIHC (**C**) and CHOL(CCA) (**D**).

We further investigated the association between its expression levels and prognosis across multiple cancers (Figure 6 and Figure S4). Data were obtained from TCGA and GTEx databases.

Results demonstrated that a low *ABCG2* expression was significantly associated with poor prognosis in KIRC and KIPAN (*p*-values < 0.01), whereas no such association was observed for LIHC or CHOL (CCA). This suggests that *ABCG2* does not directly influence liver cancer progression but may affect liver disease pathogenesis indirectly by elevating urate levels in the kidney, subsequently impacting hepatic conditions.

## 4. Discussion

Urate is often thought to be an antioxidant; however, its role varies depending on its concentration [36]. The literature shows that high concentrations of urate can lead to vascular inflammation by activating the NLRP3 inflammasome and promoting IL-1β synthesis [37]. Epidemiological studies have also found that hyperuricemia increases the risk of severe liver diseases such as fibrosis [38].

Our study demonstrates that genetically elevated SUA levels, mediated by *ABCG2* variants, have significant causal effects on hepatocarcinogenesis, particularly on CCA. The *ABCG2* gene encodes the critical transporter BCRP (breast cancer resistance protein), whose dysregulation contributes to hyperuricemia [39,40]. Clinical evidence shows that elevated SUA exacerbates treatment-related complications and impacts clinical management strategies for liver cancer patients. Early studies identified the nonsynonymous SNP rs2231142 in exon 5 of *ABCG2* as being causally linked to hyperuricemia, reducing urate transport efficiency by 54% in oocytes [41]. This is consistent with our findings, which demonstrate a causal relationship between the *ABCG2* gene variation and urate levels through Mendelian randomization.

Based on these findings, we selected SUA as a downstream biomarker for *ABCG2*. Notably, only SNPs demonstrating simultaneous associations with both SUA levels and liver diseases were included, with variants within ±100 kb of *ABCG2* coding regions, prioritized to account for potential linkage disequilibrium with the neighboring genes.

Although our findings suggest that quercetin modulates SUA levels by influencing BCRP (*ABCG2*) activity, it should be noted that the reported effects of quercetin on *ABCG2* are ambiguous and context-dependent. In vitro studies have shown that quercetin enhances the cellular accumulation and cytotoxicity of mitoxantrone (a BCRP substrate) in HeLa cells, suggesting inhibition of BCRP activity [42]. However, in mice, quercetin and its metabolite, quercetin-3-O-glucuronide, were found to upregulate renal *ABCG2* expression, thereby reducing serum urate levels [43]. In the liver, quercetin may transiently inhibit BCRP activity, potentially leading to urate retention, while chronic exposure could induce compensatory upregulation of BCRP. Notably, *ABCG2* overexpression in tumors confers chemoresistance by enhancing drug efflux, establishing it as a prognostic risk factor for treatment outcomes [44]. We therefore conclude that *ABCG2*’s role in hepatocellular carcinoma prevention by quercetin via urate modulation warrants more research.

Additionally, other mechanisms should be considered. The enzyme xanthine dehydrogenase (XDH) converts xanthine into urate [45]. Research has shown that a reduced XDH expression is associated with relatively aggressive HCC phenotypes and poor clinical outcomes, making it a potential prognostic biomarker [46]. Quercetin suppresses XDH activity, thereby reducing urate production [47]. Additionally, *XDH* has been implicated in modulating the tumor immune microenvironment, with early reports indicating positive correlations with CD8+ T-cell infiltration and negative associations with PD1+ immune cells [46]. However, the lack of liver disease-associated SNPs prevented the evaluation of *XDH* in our MR analysis framework.

Heterogeneity testing revealed significant variation in the liver and bile duct (ieu-b-4915) and NAFLD datasets. For example, in the ieu-b-4915 dataset, an MR-Egger analysis of individual SNPs demonstrated contrasting effect estimates for rs2622621 and rs2725269 (Figure S2).

Considering that heterogeneous SNPs may affect the exposure or outcome through different biological mechanisms, we retained the heterogeneous SNPs for a more comprehensive response to causal effects. Through a random-effects Inverse Variance Weighted (IVW) model, we found that heterogeneity was controllable and did not affect the causal direction. Furthermore, a leave-one-out analysis showed that excluding SNPs one by one did not lead to significant changes in the results, indicating that heterogeneous SNPs did not dominate the overall effect and that the results were robust (Figure S2).

Single-cell RNA sequencing based on GSE138709 revealed elevated *ABCG2* expression in endothelial cells of cancer patients compared to normal tissues. Immune infiltration analysis further demonstrated a positive correlation between *ABCG2* expression and endothelial cell infiltration (*p* < 0.05), a process linked to angiogenesis and enhanced tumor invasiveness. Additionally, endothelial–mesenchymal transition (EndMT) mediated by these cells may facilitate tumor cell migration and promote fibrotic microenvironments. These findings support the use of *ABCG2* overexpression as a prognostic risk factor in hepatobiliary cancers and prioritize quercetin’s preventive potential over its therapeutic potential. In the pan-cancer survival analysis, *ABCG2* showed that, unlike in the liver, a low expression of *ABCG2* in KIRC and KIPAN was associated with a poor prognosis. Since *ABCG2* is mainly expressed in the kidney, its dysregulation can increase serum urate levels, thereby affecting disease progression.

Besides urate modulation, quercetin upregulates Nrf2 protein, enhancing antioxidant enzymes (e.g., SOD, GSH), while suppressing the NF-κB pathway to mitigate oxidative stress-related pathologies such as ischemia–reperfusion injury, atherosclerosis, and cognitive impairment [48,49]. We hypothesized that quercetin or its oxidized metabolite, quercetin quinone, might bind to KEAP1 to activate Nrf2; however, no direct evidence for this was found in vitro. Early pQTL or eQTL studies also failed to identify causal links between NFE2L2 (Nrf2) and liver diseases. In our study, attempts to identify SNPs proxying NFE2L2 via downstream biomarkers were stopped due to the lack of relevant SNPs.

The Mendelian randomization method has certain limitations, such as weak instrumental variables, pleiotropy, and population stratification. These limitations can be addressed using statistical methods, such as selecting more stringent instrumental variables (*p* < 5 × 10^−8^), multiple pleiotropy evaluation methods (e.g., the Q statistic test we used), and selecting populations with the same genetic background. It should be realized that the effect estimate of exposure on the outcome obtained by MR cannot be completely equivalent to the true causal effect. It can only provide statistical clues, so further validation is critical.

For example, future studies should verify whether *ABCG2* gene knockdown mice are genetically susceptible to liver cancer and further test whether quercetin can slow the progression of liver cancer or prevent its occurrence by lowering urate levels.

Overall, this underscores the complex role of quercetin in liver cancer prevention, with effects spanning from urate transport, oxidative stress modulation, to immune regulation. While quercetin anticancer potential is supported by the findings in our study, the precise molecular mechanisms underlying its therapeutic impact still warrant further investigation.

## 5. Conclusions

We found that genetic variants in *ABCG2*, which mediate elevated SUA levels, have significant causal effects on the development of HCC, CCA, and liver fibrosis. This is consistent with previous epidemiological studies and confirms the potential of quercetin in these diseases. Additionally, *ABCG2* expression is strongly associated with endothelial cell infiltration, suggesting that *ABCG2* may contribute to enhanced tumor invasiveness. While quercetin reduces SUA levels by modulating BCRP (*ABCG2*), its tissue-specific and ambiguous effects on BCRP (*ABCG2*) indicate that more research is needed. Tentatively, we suggest that quercetin might function as a dietary supplement for preventing liver diseases, rather than as a therapeutic agent for liver cancer.

## Figures and Tables

**Table 1:** Characteristics of the GWAS cohorts.

Characteristics	GWAS-ID	Type	Sample Size	Number of SNPs
Uric acid (Urate)	ebi-a-GCST90014015	Exposure	389,404	10,783,684
Liver cell carcinoma	ieu-b-4953	Outcome	372,184	6,304,034
Liver and bile duct cancer	ieu-b-4915	Outcome	372,366	7,687,713
Malignant neoplasm of liver and intrahepatic bile ducts	finn-b-C3_LIVER_INTRAHEPATIC_BILE_DUCTS_EXALLC	Outcome	174,006	16,380,303
Chirrosis of liver	finn-b-CHIRHEP_NAS	Outcome	217,334	16,380,465
NAFLD	ebi-a-GCST90091033	Outcome	778,164	6,784,388

**Table 2:** Heterogeneity and pleiotropy analyses of urate (*ABCG2*) on liver cancer.

Disease Type	Methods	SNPs	Heterogeneity	*p*	Pleiotropy	*p*
Liver cell carcinoma (ieu-b-4953)	MR Egger	20	14.474	0.698	2.9 × 10^−5^	0.181
	IVW	20	16.409	0.630		
Liver and bile duct cancer (ieu-b-4915)	MR Egger	28	46.584	7.8 × 10^−3^	4.6 × 10^−5^	0.600
	IVW	28	47.088	9.7 × 10^−3^		
Liver and bile duct cancer (finn-b-C3_LIVER_INTRAHEPATIC_BILE_DUCTS_EXALLC)	MR Egger	46	29.846	0.949	0.040	0.692
	IVW	46	30.004	0.958		
Chirrosis of liver (finn-b-CHIRHEP_NAS)	MR Egger	46	46.033	0.388	0.033	0.840
	IVW	46	46.076	0.427		
NAFLD (ebi-a-GCST90091033)	MR Egger	40	65.087	4.0 × 10^−3^	0.010	0.607
	IVW	40	65.547	4.9 × 10^−3^		

## Data Availability

All data used in this study are available in a public repository. The code involved in the data analysis process can be obtained by contacting the corresponding author.

## Supplementary Materials

The following supporting information can be downloaded at: https://www.mdpi.com/article/10.3390/genes16040449/s1. Table S1: Retained up-regulated or down-regulated genes; Table S2: Final IVs generated from urate associated with *ABCG2* for liver cell carcinoma (ieu4953); Table S3: Final IVs generated from urate associated with *ABCG2* for liver and bile duct cancer (ieu4915); Table S4: Final IVs generated from urate associated with *ABCG2* for liver and bile duct cancer (finn-b-C3_LIVER_INTRAHEPATIC_BILE_DUCTS); Table S5: Final IVs generated from urate associated with *ABCG2* for cirrhosis of liver (finn-b-CHIRHEP_NAS); Table S6: Final IVs generated from urate associated with *ABCG2* for NAFLD (ebi-a-GCST90091033); Table S7: The SNPs used for selected gene for MR on urate; Figure S1: The Funnel Plot, Forest Plot and Leave-One-Out of ABCG2 vs. liver cell carcinoma (A), liver and bile duct cancer (B: ieu-b-4915 & C: finn-b-C3_LIVER_INTRAHEPATIC_BILE_DUCTS_EXALLC), cirrhosis of liver (D) and NAFLD (E), respectively. Figure S2: Expression levels of selected genes in the TCGA-CHOL database. From A to S the genes were *ABCG2*, *AR*, *XDH*, *CXCR1*, *UGT3A1*, *FOS*, *HSD17B2*, *TTR*, *CYP1A1*, *CXCL2*, *E2F1*, *CDK1*, *TOP2A*, *NOX4*, *NEK2*, *E2F2*, *PLK1*, *CDKN2A*; Figure S3: Immune infiltration analysis based on GSE179433. Figure S4: Pan-cancer survival analysis of *ABCG2*.
