# Supplementary material for: Reduction in Liver Cancer Risk by Quercetin via Modulation of Urate Levels: Insights from Drug-Target Mendelian Randomization
Source: Genes (Basel). 2025 Apr 13;16(4):449. doi: 10.3390/genes16040449 (PMC12027248; doi:10.3390/genes16040449)
Supplement: Supplementary file 1 [file genes-16-00449-s001.zip › genes-3511459-supplementary.pdf]

Supplementary:

Reduction in Liver Cancer Risk by Quercetin via Modulation of Urate Levels: Insights from Drug Target Mendelian Randomization

Table. S1 Retained up-regulated or down-regulated genes

Subject
Down-regulated genes
CYP1A2 TTR CXCL2 CYP1A1 ABCG2 UGT3A1 AR HSD17B2 XDH FOS CXCR1
Up-regulated genes
CASP5 CDK1 CDKN2A E2F1 E2F2 NOX4 PLK1 SQLE TERT TOP2A

Table.S2 Final IVs generated from urate associated with ABCG2 for Liver cell carcinoma(ieu4953)

Ivs	Chr	EA	ALT	β	SE	p1	p2	Variant Type
rs10022462	4	T	C	-0.0270196	4.97182e-05	1.15425e-47	6.15759e-05	intron&non_coding_transcript
rs10029588	4	A	T	0.0511742	5.16506e-05	4.45656e-154	7.01506e-05	intron
rs11736552	4	A	G	0.0433597	5.2269e-05	3.12608e-109	0.00020	downstream_gene
rs11932227	4	T	C	0.0197769	5.21732e-05	5.25412e-24	2.07101e-05	downstream_gene
rs11932727	4	T	C	-0.0283573	5.21622e-05	1.18659e-47	8.05296e-05	intergenic
rs13106683	4	T	C	-0.0458088	6.50804e-05	7.79112e-80	0.000129	intron
rs1545207	4	A	G	0.0275275	5.51571e-05	1.56459e-40	8.84665e-05	intron&non_coding_transcript
rs2622621	4	G	C	0.0699403	5.21555e-05	1e-200	0.000291	intron
rs2725221	4	A	G	0.010148	4.9604e-05	4.74111e-08	3.76165e-05	intron
rs2725269	4	T	C	0.0184655	4.96617e-05	3.10313e-23	1.71340e-05	downstream_gene
rs2728100	4	C	T	0.0811596	7.10989e-05	1e-200	6.02249e-05	intron
rs4148149	4	G	T	-0.0596188	5.21666e-05	1e-200	0.000171	intron
rs45555139	4	A	G	-0.0533074	8.34384e-05	7.54745e-67	0.000209	intron
rs4693209	4	C	G	-0.0213845	4.94698e-05	9.11801e-31	3.27721e-05	intron
rs55924362	4	G	A	-0.0317167	7.93738e-05	7.76426e-27	4.43192e-05	intron
rs59409230	4	A	C	-0.0385448	5.54673e-05	2.09942e-76	4.34407e-05	downstream_gene
rs60168325	4	C	G	0.0283078	6.59648e-05	5.20355e-30	1.95800e-05	intron&non_coding_transcript
rs7437679	4	C	A	-0.0337458	5.84881e-05	1.11944e-53	6.87951e-05	upstream_gene
rs78547757	4	C	T	-0.0223883	8.24394e-05	5.41502e-13	6.67923e-05	intergenic
rs9761468	4	A	G	-0.0266872	8.62269e-05	9.04066e-17	2.626005e-05	intergenic

Chr:chromosome;EA:effect allele;ALT: alternative allele(Other allele):*p1*： Association of SNPs with exposures； *p2*:Association of individual SNPs with outcomes

Table.S3 Final IVs generated from urate associated with ABCG2 for Liver and bile duct cancer(ieu4915)

Ivs	Chr	EA	ALT	β	SE	p1	p2	Variant Type
rs10022462	4	T	C	-0.0270196	0.00186299	1.15425e-47	6.16822e-3	intron&non_coding_transcript
rs10029588	4	A	T	0.0511742	0.00193531	4.45656e-154	3.64431e-3	intron
rs113028394	4	A	G	0.063934	0.0036603	2.56094e-68	1.19381e-3	intron&non_coding_transcript
rs113787091	4	G	T	0.0220707	0.00389761	1.4907e-08	4.06721e-3	intron
rs114314900	4	T	C	0.104632	0.00405404	6.98232e-147	5.58813e-3	intron&non_coding_transcript
rs114556806	4	T	C	-0.0350048	0.00554128	2.6652e-10	4.37502e-3	upstream_gene
rs11736552	4	A	G	0.0433597	0.00195276	3.12608e-109	7.32922e-3	downstream_gene
rs11932227	4	T	C	0.0197769	0.00195716	5.25412e-24	1.54613e-3	downstream_gene
rs11932727	4	T	C	-0.0283573	0.00195548	1.18659e-47	6.19192e-3	intergenic
rs13106683	4	T	C	-0.0458088	0.00242117	7.79112e-80	8.11725e-3	intron
rs142578439	4	G	T	0.162228	0.00472312	1e-200	6.36344e-4	intron
rs1545207	4	A	G	0.0275275	0.00206519	1.56459e-40	5.77309e-3	intron&non_coding_transcript
rs17731565	4	A	G	-0.0245008	0.0042411	7.60536e-09	4.00479e-3	intron
rs2622610	4	G	A	0.0324734	0.00388354	6.1759e-17	3.65938e-3	intron
rs2622621	4	G	C	0.0699403	0.00195697	1e-200	6.22286e-3	intron
rs2725221	4	A	G	0.010148	0.00185836	4.74111e-08	3.81966e-3	intron
rs2725269	4	T	C	0.0184655	0.00185969	3.10313e-23	1.60204e-3	downstream_gene
rs2728100	4	C	T	0.0811596	0.00268326	1e-200	7.70065e-3	intron
rs28674686	4	G	A	-0.0386349	0.00442345	2.45584e-18	4.28434e-3	upstream_gene
rs4148149	4	G	T	-0.0596188	0.0019526	1e-200	7.47285e-3	intron
rs45555139	4	A	G	-0.0533074	0.00308621	7.54745e-67	7.30509e-3	intron
rs4693209	4	C	G	-0.0213845	0.00185439	9.11801e-31	4.95919e-3	intron
rs55924362	4	G	A	-0.0317167	0.00295725	7.76426e-27	2.29454e-3	intron
rs59409230	4	A	C	-0.0385448	0.00208361	2.09942e-76	4.57279e-3	downstream_gene
rs60168325	4	C	G	0.0283078	0.0024873	5.20355e-30	2.93228e-3	intron&non_coding_transcript
rs7437679	4	C	A	-0.0337458	0.0021878	1.11944e-53	6.13009e-3	upstream_gene
rs78547757	4	C	T	-0.0223883	0.00310326	5.41502e-13	4.46920e-3	intergenic
rs9761468	4	A	G	-0.0266872	0.00320885	9.04066e-17	1.61923e-3	intergenic

Chr:chromosome;EA:effect allele;ALT: alternative allele(Other allele):*p1*： Association of SNPs with exposures； *p2*:Association of individual SNPs with outcomes(MR Egger;leave-one-out analysis)

Table.S4 Final IVs generated from urate associated with ABCG2 for Liver and bile duct cancer(finn-b-C3_LIVER_INTRAHEPATIC_BILE_DUCTS_EXALLC)

Ivs	Chr	EA	ALT	β	SE	p1	p2	Variant Type
rs10022462	4	T	C	-0.0270196	0.00186299	1.15425e-47	4.75197e-3	intron&non_coding_transcript
rs10029588	4	A	T	0.0511742	0.00193531	4.45656e-154	5.43620e-3	intron
rs112850985	4	A	G	-0.0418253	0.00684816	1.01186e-09	3.59494e-3	upstream_gene
rs113028394	4	A	G	0.063934	0.0036603	2.56094e-68	7.17151e-3	intron&non_coding_transcript
rs113617784	4	T	C	-0.0767246	0.00816274	5.48909e-21	4.49672e-3	intron
rs113787091	4	G	T	0.0220707	0.00389761	1.4907e-08	5.54175e-3	intron
rs114314900	4	T	C	0.104632	0.00405404	6.98232e-147	5.27607e-3	intron&non_coding_transcript
rs114556806	4	T	C	-0.0350048	0.00554128	2.6652e-10	4.15400e-3	upstream_gene
rs11932227	4	T	C	0.0197769	0.00195716	5.25412e-24	5.25367e-3	downstream_gene
rs11932727	4	T	C	-0.0283573	0.00195548	1.18659e-47	8.73995e-3	intergenic
rs13106683	4	T	C	-0.0458088	0.00242117	7.79112e-80	1.89593e-3	intron
rs141379351	4	C	T	0.157547	0.00926861	8.50942e-65	3.15013e-3	downstream_gene
rs141413744	4	C	T	0.0404548	0.00622171	7.91407e-11	3.40594e-3	intron
rs141505187	4	A	G	0.140224	0.00517054	5.76766e-162	5.88878e-3	intron&non_coding_transcript
rs142187387	4	G	A	-0.057485	0.00962519	2.33862e-09	4.26830e-3	intron
rs142578439	4	G	T	0.162228	0.00472312	1e-200	8.95812e-3	intron
rs144352520	4	A	G	0.115382	0.0102738	2.88204e-29	4.10827e-3	intergenic
rs1466480	4	A	G	-0.0387104	0.00337003	1.53922e-30	5.47358e-3	intron
rs149077921	4	A	G	-0.0589663	0.00888665	3.23668e-11	5.95476e-3	intron
rs151297142	4	T	G	-0.0484832	0.0068019	1.0193e-12	4.40999e-3	intron
rs1545207	4	A	G	0.0275275	0.00206519	1.56459e-40	1.31576e-2	intron&non_coding_transcript
rs17731565	4	A	G	-0.0245008	0.0042411	7.60536e-09	4.29103e-3	intron
rs189652270	4	A	G	0.0860859	0.00735107	1.12383e-31	4.01795e-3	intron&non_coding_transcript
rs2622610	4	G	A	0.0324734	0.00388354	6.1759e-17	4.48041e-3	intron
rs2622621	4	G	C	0.0699403	0.00195697	1e-200	4.54889e-3	intron
rs2725221	4	A	G	0.010148	0.00185836	4.74111e-08	5.02285e-3	intron
rs2725269	4	T	C	0.0184655	0.00185969	3.10313e-23	5.23753e-3	downstream_gene
rs2728100	4	C	T	0.0811596	0.00268326	1e-200	5.73473e-3	intron
rs28674686	4	G	A	-0.0386349	0.00442345	2.45584e-18	6.44643e-3	upstream_gene
rs4148149	4	G	T	-0.0596188	0.0019526	1e-200	8.60942e-3	intron
rs4148152	4	C	T	-0.0664979	0.00528634	2.74853e-36	6.46034e-3	intron
rs45570646	4	C	T	0.1362	0.00610883	4.0738e-110	8.08911e-3	intron
rs4693209	4	C	G	-0.0213845	0.00185439	9.11801e-31	4.38634e-3	intron
rs55924362	4	G	A	-0.0317167	0.00295725	7.76426e-27	3.78641e-3	intron
rs56233332	4	A	G	-0.0449376	0.00635809	1.57435e-12	5.13863e-3	intron
rs59409230	4	A	C	-0.0385448	0.00208361	2.09942e-76	4.51455e-3	downstream_gene
rs60168325	4	C	G	0.0283078	0.0024873	5.20355e-30	4.36825e-3	intron&non_coding_transcript
rs6847156	4	C	T	0.0435253	0.00206781	2.33776e-98	6.36506e-3	intron
rs72554039	4	A	G	-0.0703999	0.00919299	1.88843e-14	4.84135e-3	intron
rs7437679	4	C	A	-0.0337458	0.0021878	1.11944e-53	2.83851e-3	upstream_gene

rs76462878	4	T	A	-0.041979	0.0066867	3.42981e-10	5.32780e-3	intron
rs7664455	4	A	T	-0.0509495	0.00596789	1.37404e-17	5.61280e-3	downstream_gene
rs78547757	4	C	T	-0.0223883	0.00310326	5.41502e-13	3.55095e-3	intergenic
rs78948711	4	A	C	-0.0358051	0.00585109	9.39334e-10	3.67489e-3	upstream_gene
rs79931297	4	A	C	0.0844547	0.00959861	1.38484e-18	4.10071e-3	intron&non_coding_transcript
rs9761468	4	A	G	-0.0266872	0.00320885	9.04066e-17	4.89032e-3	intergenic

Chr:chromosome;EA:effect allele;ALT: alternative allele(Other allele):*p1*： Association of SNPs with exposures； *p2*:Association of individual SNPs with outcomes(MR Egger;leave-one-out analysis)

Table.S5 Final IVs generated from urate associated with ABCG2 for Chirrosis of liver(finn-b-CHIRHEP_NAS)

Ivs	Chr	EA	ALT	β	SE	p1	p2	Variant Type
rs10022462	4	T	C	-0.0270196	0.00186299	1.15425e-47	3.25323e-3	intron&non_coding_transcript
rs10029588	4	A	T	0.0511742	0.00193531	4.45656e-154	1.4703e-2	intron
rs112850985	4	A	G	-0.0418253	0.00684816	1.01186e-09	1.0780e-2	upstream_gene
rs113028394	4	A	G	0.063934	0.0036603	2.56094e-68	1.5427e-2	intron&non_coding_transcript
rs113617784	4	T	C	-0.0767246	0.00816274	5.48909e-21	1.0137e-2	intron
rs113787091	4	G	T	0.0220707	0.00389761	1.4907e-08	1.0142e-2	intron
rs114314900	4	T	C	0.104632	0.00405404	6.98232e-147	7.2877e-3	intron&non_coding_transcript
rs114556806	4	T	C	-0.0350048	0.00554128	2.6652e-10	5.4117e-3	upstream_gene
rs11932227	4	T	C	0.0197769	0.00195716	5.25412e-24	9.9852e-3	downstream_gene
rs11932727	4	T	C	-0.0283573	0.00195548	1.18659e-47	1.5710e-2	intergenic
rs13106683	4	T	C	-0.0458088	0.00242117	7.79112e-80	5.5119e-3	intron
rs141379351	4	C	T	0.157547	0.00926861	8.50942e-65	8.2698e-3	downstream_gene
rs141413744	4	C	T	0.0404548	0.00622171	7.91407e-11	1.0813e-2	intron
rs141505187	4	A	G	0.140224	0.00517054	5.76766e-162	1.4919e-2	intron&non_coding_transcript
rs142187387	4	G	A	-0.057485	0.00962519	2.33862e-09	9.8862e-3	intron
rs142578439	4	G	T	0.162228	0.00472312	1e-200	1.3848e-2	intron
rs144352520	4	A	G	0.115382	0.0102738	2.88204e-29	9.3386e-3	intergenic
rs1466480	4	A	G	-0.0387104	0.00337003	1.53922e-30	1.2935e-2	intron
rs149077921	4	A	G	-0.0589663	0.00888665	3.23668e-11	9.6335e-3	intron
rs151297142	4	T	G	-0.0484832	0.0068019	1.0193e-12	9.0914e-3	intron
rs1545207	4	A	G	0.0275275	0.00206519	1.56459e-40	1.1910e-2	intron&non_coding_transcript
rs17731565	4	A	G	-0.0245008	0.0042411	7.60536e-09	5.6711e-3	intron
rs189652270	4	A	G	0.0860859	0.00735107	1.12383e-31	8.9248e-3	intron&non_coding_transcript
rs2622610	4	G	A	0.0324734	0.00388354	6.1759e-17	1.1193e-2	intron
rs2622621	4	G	C	0.0699403	0.00195697	1e-200	6.8504e-3	intron
rs2725221	4	A	G	0.010148	0.00185836	4.74111e-08	1.0058e-2	intron
rs2725269	4	T	C	0.0184655	0.00185969	3.10313e-23	1.2200e-2	downstream_gene
rs2728100	4	C	T	0.0811596	0.00268326	1e-200	1.0152e-2	intron
rs28674686	4	G	A	-0.0386349	0.00442345	2.45584e-18	1.2115e-2	upstream_gene
rs4148149	4	G	T	-0.0596188	0.0019526	1e-200	3.6179e-3	intron
rs4148152	4	C	T	-0.0664979	0.00528634	2.74853e-36	1.3079e-2	intron

rs45570646	4	C	T	0.1362	0.00610883	4.0738e-110	9.1402e-3	intron
rs4693209	4	C	G	-0.0213845	0.00185439	9.11801e-31	4.5462e-3	intron
rs55924362	4	G	A	-0.0317167	0.00295725	7.76426e-27	7.1985e-3	intron
rs56233332	4	A	G	-0.0449376	0.00635809	1.57435e-12	1.0133e-2	intron
rs59409230	4	A	C	-0.0385448	0.00208361	2.09942e-76	1.1161e-2	downstream_gene
rs60168325	4	C	G	0.0283078	0.0024873	5.20355e-30	1.0958e-2	intron&non_coding_transcript
rs6847156	4	C	T	0.0435253	0.00206781	2.33776e-98	7.0839e-3	intron
rs72554039	4	A	G	-0.0703999	0.00919299	1.88843e-14	1.1075e-2	intron
rs7437679	4	C	A	-0.0337458	0.0021878	1.11944e-53	2.1407e-2	upstream_gene
rs76462878	4	T	A	-0.041979	0.0066867	3.42981e-10	1.2037e-2	intron
rs7664455	4	A	T	-0.0509495	0.00596789	1.37404e-17	1.0771e-2	downstream_gene
rs78547757	4	C	T	-0.0223883	0.00310326	5.41502e-13	6.8500e-3	intergenic
rs78948711	4	A	C	-0.0358051	0.00585109	9.39334e-10	1.1736e-2	upstream_gene
rs79931297	4	A	C	0.0844547	0.00959861	1.38484e-18	7.0821e-3	intron&non_coding_transcript
rs9761468	4	A	G	-0.0266872	0.00320885	9.04066e-17	8.7678e-3	intergenic

Chr:chromosome;EA:effect allele;ALT: alternative allele(Other allele):p1： Association of SNPs with exposures； p2:Association of individual SNPs with outcomes(MR Egger;leave-one-out analysis)

Table.S6 Final IVs generated from urate associated with ABCG2 for NAFLD(ebi-a-GCST90091033)

Ivs	Chr	EA	ALT	β	SE	p1	p2	Variant Type
rs10022462	4	T	C	-0.0270196	0.00186299	1.15425e-47	4.74244e-1	intron&non_coding_transcript
rs10029588	4	A	T	0.0511742	0.00193531	4.45656e-154	5.61982e-1	intron
rs112850985	4	A	G	-0.0418253	0.00684816	1.01186e-09	7.09457e-1	upstream_gene
rs113028394	4	A	G	0.063934	0.0036603	2.56094e-68	7.72900e-1	intron&non_coding_transcript
rs113617784	4	T	C	-0.0767246	0.00816274	5.48909e-21	7.78105e-1	intron
rs114314900	4	T	C	0.104632	0.00405404	6.98232e-147	6.70563e-1	intron&non_coding_transcript
rs114556806	4	T	C	-0.0350048	0.00554128	2.6652e-10	6.94572e-1	upstream_gene
rs11932227	4	T	C	0.0197769	0.00195716	5.25412e-24	7.43412e-1	downstream_gene
rs11932727	4	T	C	-0.0283573	0.00195548	1.18659e-47	5.70427e-1	intergenic
rs13106683	4	T	C	-0.0458088	0.00242117	7.79112e-80	5.02549e-1	intron
rs141413744	4	C	T	0.0404548	0.00622171	7.91407e-11	7.82817e-1	intron
rs141505187	4	A	G	0.140224	0.00517054	5.76766e-162	7.86406e-1	intron&non_coding_transcript
rs142578439	4	G	T	0.162228	0.00472312	1e-200	8.84921e-1	intron
rs1466480	4	A	G	-0.0387104	0.00337003	1.53922e-30	7.29962e-1	intron
rs149077921	4	A	G	-0.0589663	0.00888665	3.23668e-11	7.72394e-1	intron
rs151297142	4	T	G	-0.0484832	0.0068019	1.0193e-12	7.47663e-1	intron
rs1545207	4	A	G	0.0275275	0.00206519	1.56459e-40	5.15060e-1	intron&non_coding_transcript
rs17731565	4	A	G	-0.0245008	0.0042411	7.60536e-09	7.72192e-1	intron
rs189652270	4	A	G	0.0860859	0.00735107	1.12383e-31	6.17060e-1	intron&non_coding_transcript
rs2622610	4	G	A	0.0324734	0.00388354	6.1759e-17	7.07789e-1	intron

rs2622621	4	G	C	0.0699403	0.00195697	1e-200	6.88252e-1	intron
rs2725221	4	A	G	0.010148	0.00185836	4.74111e-08	8.23546e-1	intron
rs2725269	4	T	C	0.0184655	0.00185969	3.10313e-23	7.98587e-1	downstream_gene
rs2728100	4	C	T	0.0811596	0.00268326	1e-200	9.10102e-1	intron
rs28674686	4	G	A	-0.0386349	0.00442345	2.45584e-18	7.39399e-1	upstream_gene
rs4148149	4	G	T	-0.0596188	0.0019526	1e-200	5.94232e-1	intron
rs4148152	4	C	T	-0.0664979	0.00528634	2.74853e-36	8.71310e-1	intron
rs45570646	4	C	T	0.1362	0.00610883	4.0738e-110	9.83748e-1	intron
rs4693209	4	C	G	-0.0213845	0.00185439	9.11801e-31	7.38774e-1	intron
rs55924362	4	G	A	-0.0317167	0.00295725	7.76426e-27	7.44061e-1	intron
rs56233332	4	A	G	-0.0449376	0.00635809	1.57435e-12	6.72029e-1	intron
rs59409230	4	A	C	-0.0385448	0.00208361	2.09942e-76	9.02723e-1	downstream_gene
rs60168325	4	C	G	0.0283078	0.0024873	5.20355e-30	7.42619e-1	intron&non_coding_transcript
rs72554039	4	A	G	-0.0703999	0.00919299	1.88843e-14	7.80815e-1	intron
rs7437679	4	C	A	-0.0337458	0.0021878	1.11944e-53	6.98837e-1	upstream_gene
rs76462878	4	T	A	-0.041979	0.0066867	3.42981e-10	7.60052e-1	intron
rs78547757	4	C	T	-0.0223883	0.00310326	5.41502e-13	7.64801e-1	intergenic
rs78948711	4	A	C	-0.0358051	0.00585109	9.39334e-10	7.49586e-1	upstream_gene
rs79931297	4	A	C	0.0844547	0.00959861	1.38484e-18	7.62407e-1	intron&non_coding_transcript
rs9761468	4	A	G	-0.0266872	0.00320885	9.04066e-17	7.47026e-1	intergenic

Chr:chromosome;EA:effect allele;ALT: alternative allele(Other allele):p1： Association of SNPs with exposures；p2:Association of individual SNPs with outcomes(MR Egger;leave-one-out analysis)

Table.S7 The SNPs used for selected gene for MR on urate

Genes	Ivs	Chr	EA	ALT	β	SE	p1	p2	Variant Type
ABCG2	rs56317495	4	G	A	0.361565	0.0263317	6.60237e-43	4.59544e-8	downstream_gene
	rs62308057	4	G	C	0.589154	0.0169925	1e-200	5.51184e-32	downstream_gene
CASP5	rs117527815	11	G	T	-0.163918	0.0255707	1.44951e-10	7.49311e-1	intron
	rs1910398	11	C	T	0.720786	0.0205558	1e-200	9.66459e-1	intergenic
	rs477043	11	C	T	0.194091	0.0355765	4.88079e-08	7.51388e-1	intron
	rs542720	11	C	A	0.581681	0.0184896	1e-200	9.24009e-1	intron
	rs581364	11	C	T	-0.221951	0.0117905	4.73042e-79	7.35876e-1	intergenic
CXCR1	rs4672870	2	T	C	-0.448906	0.0114468	1e-200	0.08870415	intron
	rs62183956	2	T	C	0.451272	0.011284	1e-200	0.2862167	downstream_gene
E2F2	rs114352248	1	T	C	0.167078	0.0223143	7.02587e-14	0.3508512	intron
	rs11576825	1	G	C	0.177614	0.0239414	1.18359e-13	0.6558292	regulatory_region
SQL2	rs10103274	8	G	A	0.353295	0.0207888	9.03441e-65	0.2291506	intron
	rs7821961	8	G	C	0.105061	0.0181786	7.4967e-09	0.3812061	intron&non_coding_transcript
PLK1	rs35586	16	G	A	0.290065	0.0140427	8.65765e-95	0.8834806	synonymous
	rs8049250	16	G	C	-0.350091	0.0440017	1.77133e-15	0.837242	upstream_gene

Chr:chromosome;EA:effect allele;ALT: alternative allele(Other allele):p1：Association of SNPs with exposures；p2:Association of individual SNPs with outcomes(Wald ratio)

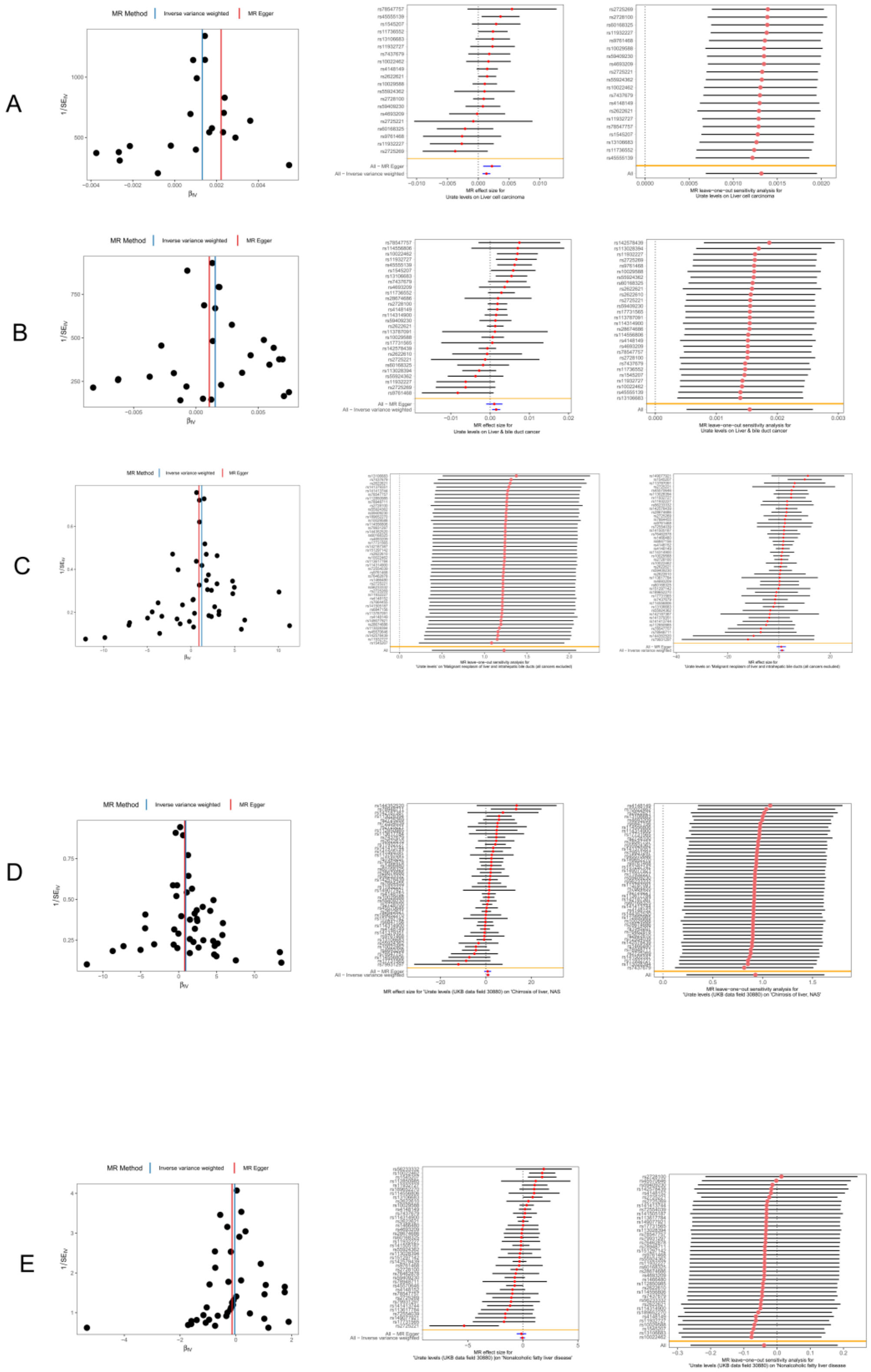

Figure S1 The Funnel Plot,Forest Plot and Leave-One-Out of *ABCG2* vs Liver cell carcinoma(A),Liver and bile duct cancer(B:ieu-b-4915&C:finn-b-C3_LIVER_I

NTRAHEPATIC_BILE_DUCTS_EXALLC), Chirrosis of liver(D) and NAFLD(E), respectively.

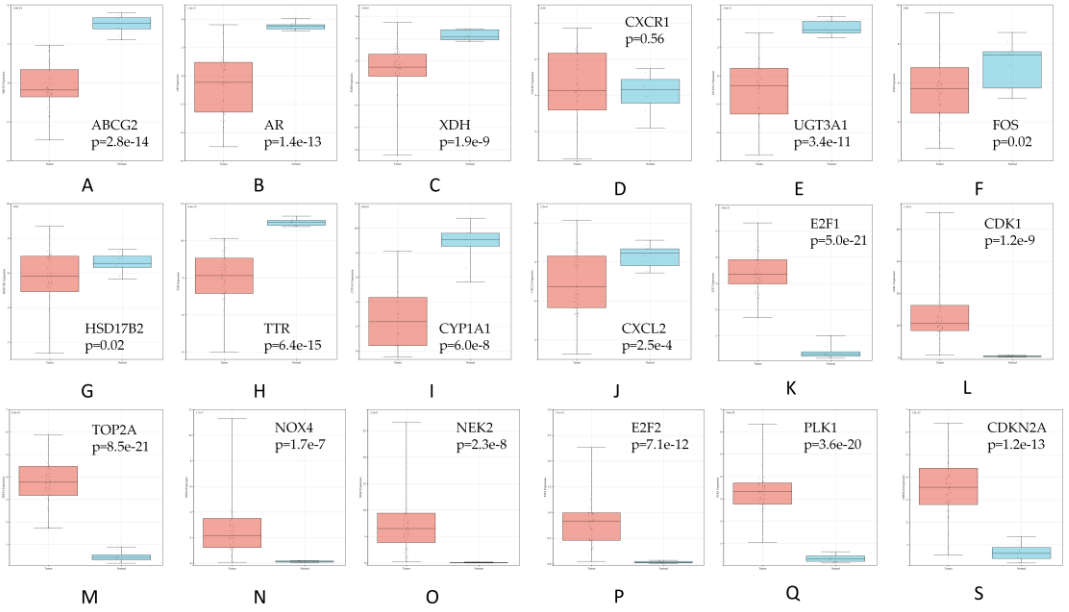

Figure S2 Expression levels of selected genes in the TCGA-CHOL database. From A to S were: *ABCG2,AR,XDH,CXCR1,UGT3A1,FOS,HSD17B2,TTR,CYP1A1,CXCL2,E2F1,CDK1,TOP2A,NOX4,NEK2,E2F2,PLK1,CDKN2A*.Where Blue represented the Normal group and Red represented the Tumor group.

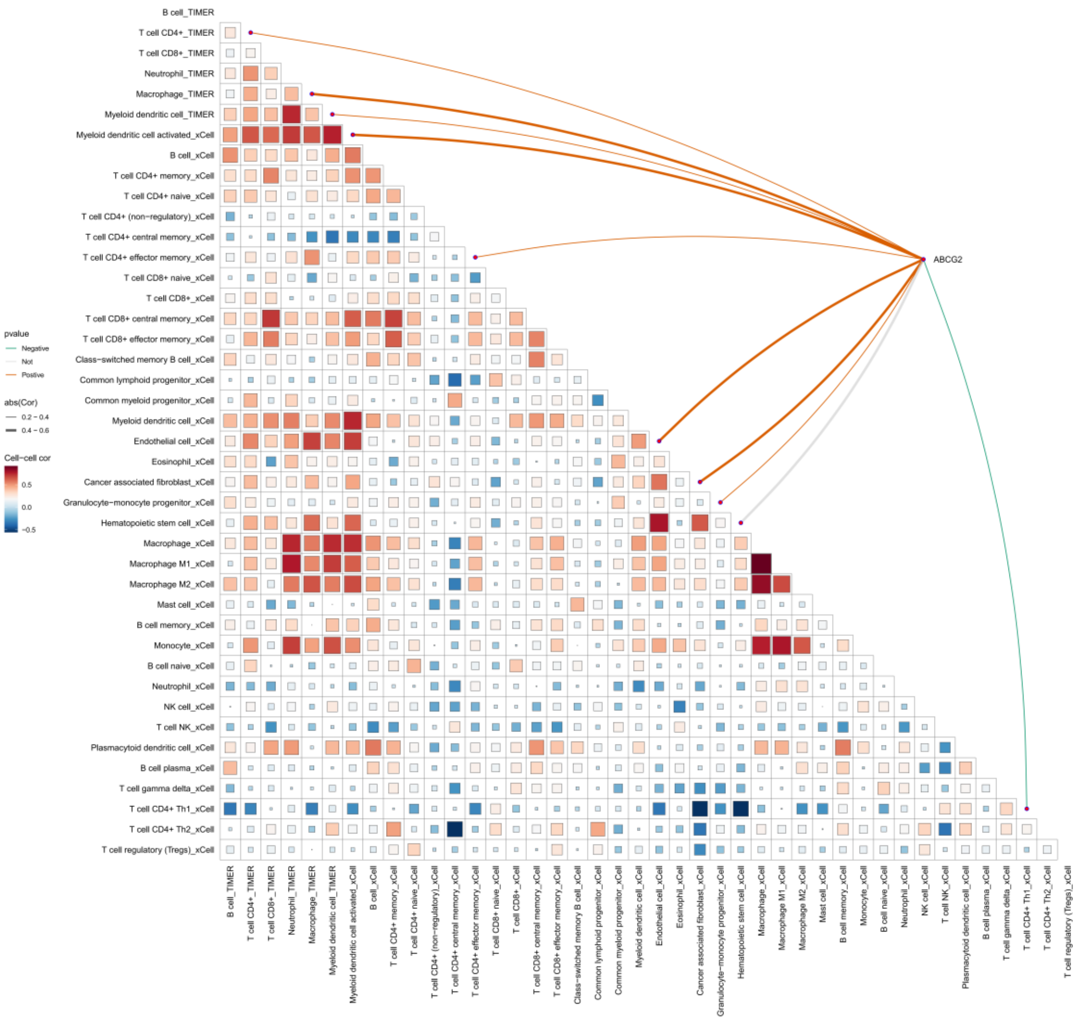

Figure S3 Immune infiltration analysis Based on GSE179443

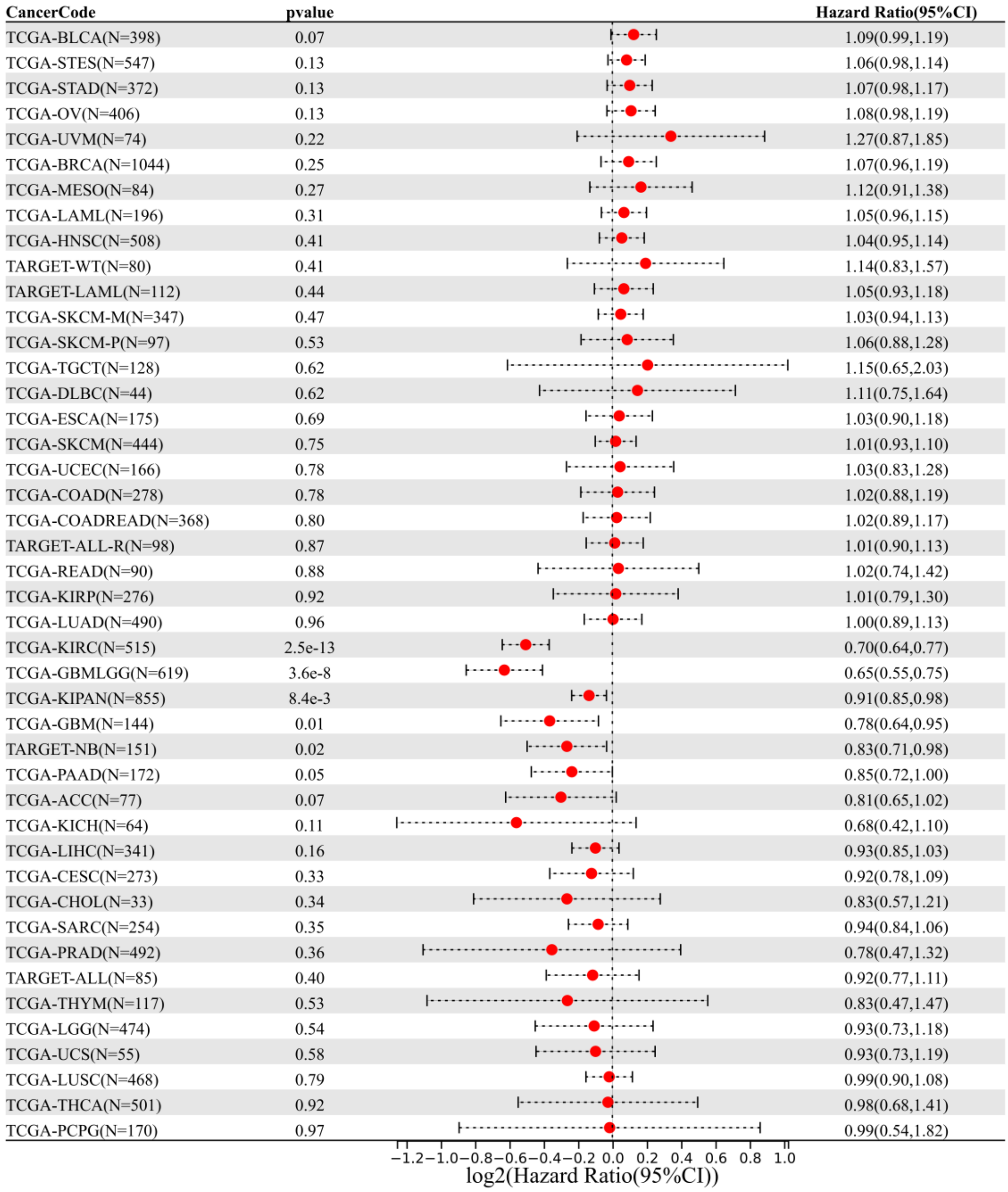

Figure S4 Pan-cancer survival analysis of *ABCG2*
